# Manual of Practical Medical and Physiological Chemistry

**Published:** 1893-02

**Authors:** 


### Book Reviews.

MANUAL OF PRACTICAL MEDICAL AND PHYSIOLOGICAL CHEMISTRY, by Charles E. Pellew, E. M., Demonstrator of Physics and Chemistry in the College of Physician's and Surgeons, etc. D. Appleton & Co., N. Y.

THE author endeavors to give us a manual of chemistry, prepared especially for the student of medicine. The student is expected to have some knowledge of general chemistry, and to be familiar with at least the fundamental principles of chemical philosophy, and should have had some experience in laboratory manipulation.

Part I. takes up the *carbohydrates* and is divided into five lessons, each lesson giving the occurrence, preparation, properties and uses of the substance treated of, and closes with laboratory experiments, giving tests for detection and recognition of substances and, when necessary, microscopic appearances with plates.

Part II. is divided into two lessons, and treats of the fats and fixed oils.

Part III. has three lessons devoted to the *Proteids* and *Albuminous* bodies, with laboratory experiments, showing tests for the various proximate principles of the human body.

Part IV. treats of the inorganic constituents of the body.

Part V. is devoted to water analysis.

Part VI. takes up the animal tissues and secretions and contains four valuable lessons, one of which is devoted to the consideration of milk, giving special attention to the tests for *breast* milk.

Part VII. treats of the subject of *digestion*, giving laboratory experiments on gastric, pancreatic and salivary digestion.

Parts VIII. and IX. are devoted to the examination of urine, special attention being given the microscopical examination of urinary sediments. The plates are numerous throughout the book, and are unusually good; indeed, the general make up of the work is first-class, both paper and printing being of superior quality. L. H. J.

